# Matching IRT Models to Patient-Reported Outcomes Constructs: The Graded Response and Log-Logistic Models for Scaling Depression

**DOI:** 10.1007/s11336-021-09802-0

**Published:** 2021-08-31

**Authors:** Steven P. Reise, Han Du, Emily F. Wong, Anne S. Hubbard, Mark G. Haviland

**Affiliations:** 1 Department of Psychology, University of California, Los Angeles, Los Angeles, USA; 2 Department of Psychiatry, Loma Linda University, Los Angeles, USA

**Keywords:** graded response model, log-logistic model, IRT model assumptions

## Abstract

Item response theory (IRT) model applications extend well beyond cognitive ability testing, and various patient-reported outcomes (PRO) measures are among the more prominent examples. PRO (and like) constructs differ from cognitive ability constructs in many ways, and these differences have model fitting implications. With a few notable exceptions, however, most IRT applications to PRO constructs rely on traditional IRT models, such as the graded response model. We review some notable differences between cognitive and PRO constructs and how these differences can present challenges for traditional IRT model applications. We then apply two models (the traditional graded response model and an alternative log-logistic model) to depression measure data drawn from the Patient-Reported Outcomes Measurement Information System project. We do not claim that one model is “a better fit” or more “valid” than the other; rather, we show that the log-logistic model may be more consistent with the construct of depression as a unipolar phenomenon. Clearly, the graded response and log-logistic models can lead to different conclusions about the psychometrics of an instrument and the scaling of individual differences. We underscore, too, that, in general, explorations of which model may be more appropriate cannot be decided only by fit index comparisons; these decisions may require the integration of psychometrics with theory and research findings on the construct of interest.

**Supplementary Information:**

The online version supplementary material available at 10.1007/s11336-021-09802-0.

## Introduction

Item response theory (IRT) models were developed to solve practical testing problems in large-scale, multiple-choice, cognitive aptitude testing (see Lord, 1980). IRT models and the psychometric procedures derived from them have brought about revolutionary changes in how cognitive ability tests are analyzed, developed, administered, and scored. Common applications include: (a) linking methods to place test scores from different item sets onto the same scale (Lee & Lee, 2018), (b) statistical approaches for detecting differential item functioning to identify items that may be inappropriate for some examinee populations (Millsap, 2012), and (c) computerized adaptive testing methods for achieving precise scores with as few items as possible (Magis, Yan, & von Davier, 2017).

Over the past two decades, applications of parametric IRT    models have been extended beyond multiple-choice ability testing to, for example, the domains of political science (Treier & Jackman, 2008), sociology (Osgood, McMorris, & Potenza, 2002), personality (Steinberg & Thissen, 1995; Reise & Waller, 1990), psychopathology (Thomas, 2011; Reise & Waller, 2003), attitudes (Reiser, 1981), health-related quality of life (HRQoL; Cella, Chang, & Heinemann, 2002), and patient-reported outcomes (PRO; Chang & Reeve, 2005), most notably in the National Institutes of Health-funded Patient Reported Outcomes Measurement Information System project (PROMIS®; Cella et al., 2007; Reeve, Hays, Bjorner, et al., (2007).

A neglected matter of importance, however, is the use of IRT models in domains outside multiple choice, cognitive ability testing, for in general, IRT models have not been adapted to different types of constructs. The application of IRT models in HRQoL and PRO, for example, consists almost exclusively of using existing methods and procedures regardless of a construct’s characteristics (e.g., DeWitt et al., 2011; Reeve et al., 2007). In the following section, we consider some of the explicit and implicit assumptions underlying standard IRT model applications and how HRQoL and PRO constructs and their associated measures may violate these assumptions.^1^

We then examine a specific model, the log-logistic (LL; Lucke, 2013; 2015), which was proposed to provide an alternative measurement model for specific types of unipolar, PRO constructs (addictive disorders). Our primary goal is to detail how the LL model differs from a traditional graded response model (GRM; Samejima, 1969) in terms of: (a) the functional form of the item response curve, (b) the instrument’s psychometrics, (c) interpreting the meaning of the trait scale, and (d) evaluating measurement precision. We will not argue that the LL model “fits better” or is “more valid” than the GRM. Rather, our objective is limited to demonstrating that two, essentially equivalent models, can yield very different psychometric portraits of an instrument and different scalings of individual differences. In evaluating these portraits, we underscore the role that psychological theory and research must ultimately play in the selection of measurement models.

### IRT model assumptions

Valid applications of commonly used parametric IRT models stand on three fundamental assumptions (Embretson & Reise, 2000). The first, “monotonicity,” is that as trait levels increase, the expected score on the item (i.e., the item response curve) increases. This assumption is required because logistic IRT models are monotonically increasing, so for the model to be valid, item endorsement rates must increase as trait levels increase.

The second and third assumptions, unidimensionality and local independence, although distinct, are highly inter-related (see Ip, 2010); each has its own statistics to determine whether the assumption has been met. Unidimensionality is achieved when the covariance among the test items is explained by a single, common factor (see Bonifay, Reise, Scheines, & Meijer, 2015, for a nuanced explanation). The term “common” is critically important because for any scale item, multiple systematic factors cause variance in item responses. The partitioning of variance in the common factor model is written as:

1 $$\begin{aligned} \hbox {Var(Item) }= \hbox {Var(Common)} + \hbox {Var(Specific to Item)} +\hbox { Unique (Random)} \end{aligned}$$

Equation 1 states that the variance of an item response is a function of a common factor (or factors) shared with other items, plus variance due to systematic/reliable sources unique to the item (item specific variance), plus variance due to unsystematic/unreliable sources.

Appropriately, some researchers describe unidimensionality as a single common factor explaining item inter-relations. Under this correct interpretation, when unidimensionality is reasonably met, estimated item parameters reflect the relation between the item properties and the single common factor on which the researcher expects to scale people. Moreover, in unidimensional data, it does not matter which content items are administered as trait level estimates would remain relatively unbiased. When unidimensionality is violated, it is not clear what combination of common factors the latent variable reflects, and estimated item parameters may change radically depending on what subset of items are included or excluded during item parameter calibration.

We caution that the interpretation of unidimensionality is sometimes mischaracterized in the applied literature. Some researchers, for example, claim that tests of unidimensionality determine whether the construct itself is a “unidimensional construct” or a “multidimensional construct.” These oft-repeated assertions are misleading, as constructs are theoretical ideas that do not have statistical dimensionalities. van der Mass et al. (2011, p. 353), writing about the reification of latent variables and the conflation of statistical (uni)dimensionality with psychological reality, aptly make this point about fitting a unidimensional IRT model:

However, this should not be mistaken for evidence that a single ability is in play. It merely means that individual differences in performance can be reasonably described by a scalar variable. Statistical unidimensionality, thus, does not imply that a single ability is measured.

Local independence is achieved when, controlling for trait level(s), the items are statistically independent. There may be only a single, strong common factor underlying item responses (unidimensionality), but pairs of items can display local dependence (i.e., show residual correlations after controlling for the common trait) due to their including common words, covering the same content but phrased in slightly different ways (“I like to attend parties,” “parties can be fun to attend”), or even due to residual factors caused by reverse-scored questions. Typically, local dependence violations cause the slope parameters to be inflated because such item pairs have inflated correlations and, thus, the latent variable is “pulled” toward the redundant item pair rather than the common factor underlying all items (see also, Tuerlinckx & De Boeck, 2001, for more details).

These are the three fundamental assumptions, but for a model application to make clear sense, there are other implicit, but equally important assumptions, as Reise, Rodriguez, Spritzer, and Hays (2018) note:

To be used effectively, however, models such as the logistic GRM (Samejima, 1969) make many assumptions about the latent trait (causative, not emergent), the item response data (local independence), the calibration sample (homogeneous, representative), the nature and shape of the latent variable (continuous, normal), the distribution of errors, and the parametric form of the model (linear relation between theta and log-odds of responding). The validity of the conclusions drawn from any IRT model application is threatened to the degree that any of these assumptions are violated. (p. 372)

We do not question that PRO data can *approximately* meet the first three explicit (logistic) IRT model assumptions—monotonicity, unidimensionality, and local independence. We, however, do question whether PRO data are consistent with some of the implicit assumptions, for example, continuous bipolar trait and normality, as we explain next.

### Standard IRT Models and PRO Constructs

We describe five potentially important differences between measuring ability constructs (e.g., verbal ability) versus PRO (or HRQoL) constructs (e.g., depression, pain intensity, fatigue, upper body mobility). Among these differences are: (a) non-normal latent variables, (b) unipolar constructs, (c) the quasi-continuous nature of the latent trait metric, (d) the nomothetic applicability of the construct, and (e) the presence of excess zeros. Clearly, these five properties, which are particularly salient in PRO measurement, are by no means independent; nevertheless, for the purposes of clarity, we describe each separately.

*Normality* Traits such as verbal and math ability may well be reasonably normally distributed in the population; however, for many non-cognitive constructs involving psychopathology (e.g., anger), limits in functioning (e.g., upper body mobility), physical challenges (e.g., chronic pain), and addictions (e.g., excessive alcohol use), one would not expect normal distributions in the general population. Although specifying an IRT model does not imply any particular distribution for the latent trait, IRT item parameters, typically, are estimated assuming a normal distribution for the latent trait. If IRT models are estimated, or the fit of a one factor model is evaluated using limited information factor analytic methods, tetrachoric or polychoric correlations will form the basis of the analyses. Both tetrachorics and polychorics assume a continuous underlying normal distribution.

IRT parameter estimates are commonly based on a marginal maximum likelihood full information solution, such as the R library *mirt* (Chalmers, 2012). In this estimation, the method of quadrature is used to specify a normal latent trait distribution that is fixed to estimate the item parameters. Although programs such as *mirt* allow one to estimate an “empirical histogram” latent trait distribution (Mislevy, 1984; Woods, 2007) based on sample data instead of relying on the assumed normal population, these methods have not been shown to work reliably under a variety of conditions and, thus, are seldom used. A fair amount has been written about the detrimental effects (e.g., biased item parameter estimates) of fitting IRT models when the underlying latent variable is not normal (e.g., Monroe & Cai, 2014; Woods & Thissen, 2006). Although alternative models for handling non-normality have been proposed (reviewed by Reise & Rodriguez, 2016; Reise et al., 2018), none is applied routinely, and we argue that the problems in fitting IRT models to PRO constructs due to latent trait distributional issues extend well beyond adjusting the parameter estimates for “non-normality,” as we describe next.

*Unipolar Traits* Typically, trait level estimates for constructs, such as verbal or mathematical ability, are interpreted as reflecting relative standing (i.e., distance above or below a mean) along a continuous dimension, and they are bipolar (both ends of the scale are meaningful). Fully continuous trait scales for ability constructs imply that it should be possible to construct items that distinguish between individuals anywhere along the trait continuum from low to medium to high trait levels. Finally, ability traits generally are thought of as nomothetic in that they apply to the general population, including both “normal” community samples and special subgroups of the population.

In contrast, many PRO constructs are unipolar; that is, the trait is meaningful at one end of the distribution but not the other. Reise and Waller (1990) pointed this out over 30 years ago in their evaluation of IRT models fitted to scales from the Multidimensional Personality Questionnaire (Tellegen, 1982). After observing the extremely peaked information curves in these “normal” range personality measures, they questioned, for example, what it meant to be low on constructs such as alienation or how one could write items that provided information in low ranges of the alienation continuum. Analogously, low levels of depression do not differentiate among different gradations of elation, happiness, or joy; they merely represent an absence of depressive symptoms. Other constructs and associated measures—pain, fatigue, and alcoholism—are also unipolar. This can profoundly affect an IRT application because the model should in some way recognize that low scores reflect the absence of a quality and not a relatively low score “below the mean.”

*Quasi-Continuous* Related to the unipolar construct issue is the quasi-continuous nature of the latent trait. In IRT models, the latent trait scale ranges from -infinity to $+$infinity, and it makes sense that everyone has a position on the scale. Yet, clearly, for unipolar constructs, the notion of meaningful high and low scores is problematic. One hallmark of such quasi-continuous constructs is the inability of researchers to write items that provide discrimination across the trait range. As reviewed in Reise and Waller (2009), for many psychopathology and normal-range personality constructs, even when using polytomous multi-point items, threshold (or location) parameters are bunched together at one end of the scale; that is, they are not spread out over the range as one might expect. This is a hallmark characteristic of quasi-continuous traits (Reise & Waller, 2009; Reise et al., 2018). A related occurrence, commonly observed in PRO data, is a distribution of IRT scores that display either a ceiling or floor effect (Hays et al., 2016) as there are no items that provide information at one end of the latent trait and, thus, there is no way to spread out the trait level estimates.

*Special Populations and Excess Zeros* Finally, the fourth and fifth differences are highly inter-related, as well as related to the unipolar nature of the constructs. Specifically, some PRO constructs are only applicable to a limited subset of the population. Consider Lucke’s (2015) example of gambling addiction. Measuring addiction severity is only relevant to people who gamble or who can gamble. In other words, the construct does not necessarily apply to everyone in the world—not everyone can be placed on a relative standing “continuum.” Constructs such as pain (after a particular procedure), and fatigue (after chemotherapy) are also most relevant to specific clinical populations; thus, attempts to develop a metric on which the “general” population can be scaled and compared may not be appropriate. Nevertheless, many researchers want a scale that generalizes to an entire community, so they include both people-in-general and clinical cases in the calibration sample and scoring. This practice often leads to an “excess zeros” problem, which occurs when one mixes non-case and case data. The item slopes become artificially high (see Reise et al., 2018); the more non-cases are over sampled, the more IRT slope parameters are inflated. The occurrence of excess zeros also presents challenges for scale linking because slopes may differ across populations due to different case mixes, not merely mean and variance on the latent trait differences.

### Alternative IRT-Related Models

The distinct features of PRO constructs and measurement challenges have not escaped the attention of psychometricians (especially in psychiatric measurement), and, indeed, alternative models have been developed to address one or more of the above-raised concerns. For example, methods of estimating the shape of the latent trait distribution based on Ramsay curves (Woods, 2006; 2015; Woods & Thissen, 2006), as well as heteroskedastic-skew models (Molenaar, Dolan, & de Boeck, 2012), have been developed to address the challenges of accurately estimating item parameters in the presence of non-normal distributions. Note, however, that in these models, the unique contribution is the estimation of the latent distribution alongside the estimation of the item response curves; the fitted model still is the traditional logistic graded response model.

An interesting development is the estimation of zero-inflated mixture models (e.g., Finkelman, Green, Gruber, & Zaslavsky, 2011) designed to handle IRT modeling when the population is heterogeneous (cases vs. non-cases) and the continuous trait (e.g., depression) is considered to be applicable to only a subset of the population. Most recently, for example, Magnus and Garnier-Villarreal (2021) proposed a zero-inflated multidimensional graded response model. Their approach consists of estimating each individual’s standing on two correlated latent variables, one representing “susceptibility to the construct” and the other “severity.”

Further, Wall, Park and Moustaki (2015) demonstrated the use of a zero-inflated mixture graded response model with Mplus. This model treats zero and near-zero scores as a distinct latent class and then estimates IRT item parameters with a normality assumption only for a “traited class.”^2^ The authors justify the model’s development (p. 583), “It has been argued that standard IRT models of psychiatric disorder symptoms may be problematic due to the unipolar nature of many clinical traits. In the current article, the authors propose to address this by using a mixture model to approximate the unknown latent trait distribution in the IRT model while allowing for the presence of a nonpathological subgroup.”

Finally, and most relevant to the present demonstration, Magnus and Liu (2018) proposed a zero-inflated LL model (Lucke, 2013; 2015). The LL model is particularly noteworthy because it was proposed to address the unipolar trait issue. Similar to Wall, Park, and Moustaki’s (2015) work, these authors justify their model by referring to the special nature of psychiatric constructs (e.g., on p. 571): “This research introduces a latent class item response theory (IRT) approach for modeling item response data from zero-inflated, positively skewed, and arguably unipolar constructs of psychopathology.”

Each of these developments shows great promise. Thoughtful critiques of these models, however, are beyond the scope of the present paper. Rather, for demonstration purposes, we apply a model specifically designed to address the unipolar trait problem, namely the LL model described by Lucke (2013; 2015) and implemented in Magnus and Liu (2018). The LL is particularly relevant here because, as we show, it can provide a near equivalent model to the graded response model but yield a fundamentally different interpretation of the psychometrics and scaling individual differences. We do not use the zero-inflated version of the LL model in this demonstration, because applying the log-logistic to all individuals in our sample is more consistent with what is routinely done in PRO measurement—treating all individuals, whether from clinical or community samples, as “scalable” on the depression dimension.

### Present Research

The objective of the present research is to demonstrate that fitting a LL model can provide very different psychometrics, relative to the standard GRM. To accomplish this, we will interpret both the LL model and the traditional GRM, when applied to an 8-item polytomously scored, self-report, short form measure of depression (items listed in Table 1). The response options are: *never *(0), *rarely *(1), *sometimes *(2), *often *(3), and *always *(4), and the time frame is occurring in the past 7 days. The *n* was 3,000 adults sampled to match the general population, collected as part of an internet survey (http://op4g.com/our-panel/) for the PROMIS®project (see Hays et al., 2016 for demographic details). We selected this data set for several reasons. First, PROMIS®measures were developed after an exhaustive search of the depression measurement literature, followed by a careful winnowing down and refinement of item content, and empirical comparisons to existing gold-standard measures (e.g., see DeWalt et al., 2007; Kelly et al., 2011; Pilkonis et al., 2011). We, thus, are quite confident that this is a well-constructed measure. Second, depression is an excellent example of a unipolar construct. The low end does not represent any substantive construct but rather the absence of depression symptoms. Our application differs from Lucke’s, however, in that he had a specific theory about addictive disorders and why symptom counts would be exponentially related to trait levels. In turn, this substantive theory of addiction was used to justify the specification of the LL model and the assumed log normal distribution for the latent trait. In the present demonstration, we do not incorporate an explicit theory of depression to justify one model over the other (i.e., logistic versus LL). We will return to the need for theory and related topics in the discussion.

**Table 1:** PROMIS depression item content

Item	Content
1	I felt worthless
2	I felt that I had nothing to look forward to
3	I felt helpless
4	I felt sad
5	I felt like a failure
6	I felt depressed
7	I felt unhappy
8	I felt hopeless

## Psychometrics: GRM and LL

We begin by describing the data from a classical test theory framework. In Table 2 are shown *r.drop* (item-test correlation after dropping the item from the total score) and item mean. Item-test correlations are very high, averaging .85 (*SD* $=$ 0.1). The average item intercorrelation is .75, and coefficient alpha is .96. The two “easiest” items to endorse (high means) are #4 (*I felt sad*) and #7 (*I felt unhappy*), whereas the most “difficult” (lowest means) items are #1 (*I felt worthless*) and #8 (*I felt hopeless)*.

**Table 2:** Item-scale correlations (corrected for item overlap), means, and response proportions

Item	R.drop	Mean	Response proportions				
			0	1	2	3	4
1	.83	1.05	.47	.21	.18	.09	.05
2	.84	1.20	.42	.20	.21	.11	.06
3	.85	1.21	.40	.21	.22	.12	.05
4	.83	1.44	.28	.25	.28	.14	.06
5	.87	1.15	.43	.20	.21	.10	.06
6	.85	1.33	.35	.22	.24	.14	.06
7	.84	1.43	.28	.25	.28	.13	.06
8	.87	1.14	.44	.19	.20	.12	.05
Mean	.85	1.24	.38	.22	.23	.12	.06
SD	0.01	0.05	.07	.02	.03	.02	.00

R.drop is itemtest correlation after dropping item score from composite.

**Fig. 1:**
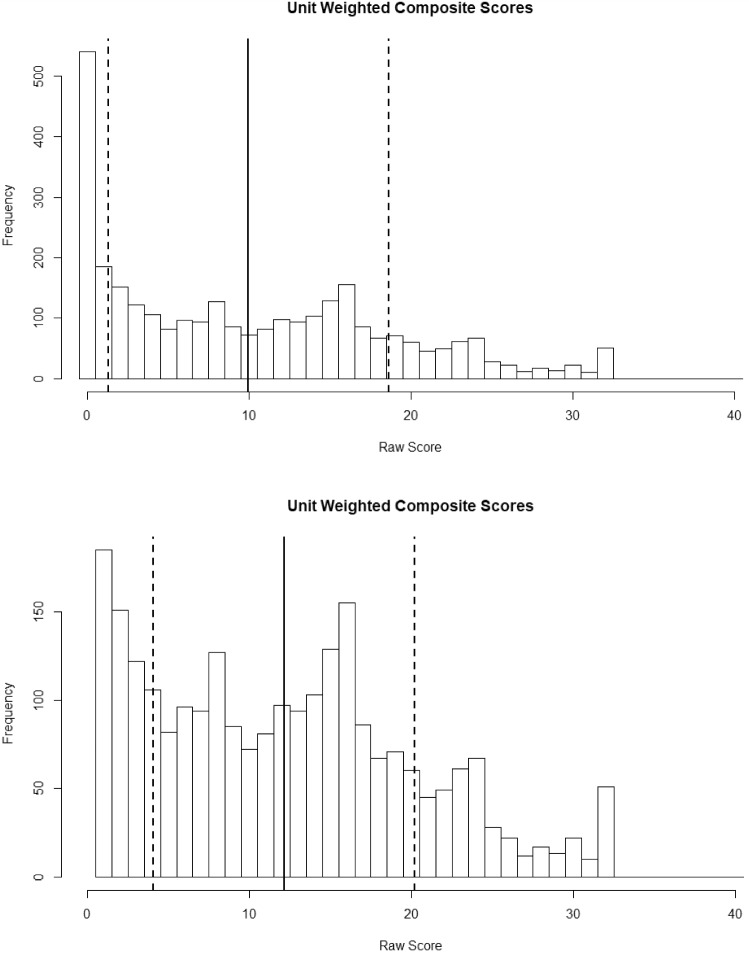
Histograms of raw score distributions include (top) and excluding (bottom) all zero response patterns.

In the top of Fig. 1 is displayed a histogram of unit-weighted composite scores, which is unsurprisingly highly positively skewed, with many all zero response patterns (540/3000 or 18%). The solid line is the mean of 9.95, and the dashed lines are plus and minus one standard deviation (8.66). Clearly, it is not possible to score two or three standard deviations below the mean on this measure. The minimum raw score $=$ 0, 1st quartile $=$ 2, median $=$ 8, mean $=$ 9.95, 3rd quartile $=$ 16, and maximum $=$ 32. In the bottom of Figure 1 is displayed the same histogram, but without the zero scores so that the distribution in the non-zero “symptomatic” group is clearer. In this figure, it appears that raw scores have a half normal distribution.^3^ The minimum raw score $=$ 1, 1st quartile $=$ 5, median $=$ 12, mean $=$ 12.14, 3rd quartile $=$ 17, and max $=$ 32.

### The Graded Response Model

A commonly applied unidimensional IRT model for polytomous item responses is the GRM. For the present 5-category data, in the GRM, for each item there will be the number of response options (5) minus 1 ($= 4$), threshold response curves (TRCs). These TRCs for item *j* describe the probability of responding above between category threshold *k* ($k = 1\ldots 4$) conditional on the latent trait:

2 $$\begin{aligned} P_{jk}^{*}(Y\le k\vert \theta )= \frac{1}{1+\mathrm {exp}(-\alpha _{j}\left( \theta -\beta _{jk} \right) )} \end{aligned}$$

where $\theta$ is a continuous latent variable from to $+$ infinity, assumed to underlie the item responses. To set the scale for the latent variable and, thus, the item parameters, the mean of the latent variable, typically, is fixed to 0 with a variance of 1; the metric, thus, looks like the familiar z-score scale. The $\alpha _{j}$ parameter is the item slope or “discrimination” reflecting the steepness of the ($k - 1 = 4$) TRCs at the inflection point (higher slopes reflect greater discrimination). The ($k - 1 = 4$) $\beta _{jk}$ parameters are the item locations indicating the point on the latent trait scale where the probability of responding above a given between category boundary is .50. Positive values reflect more severe, rarely endorsed categories, and negative values reflect category responses that require lower trait standing.

The conditional probability of responding in each category is found by subtracting adjacent $P_{jk}^{*}\vert \theta$ as follow:

$$\begin{aligned}&P(x=0)\vert \theta =1- P_{j1}^{*}\vert \theta \quad P(x=1)\vert \theta =P_{j1}^{*}\vert \theta - P_{j2}^{*}\vert \theta \\&P(x=2)\vert \theta =P_{j2}^{*}\vert \theta - P_{j3}^{*}\vert \theta \quad P(x=3)\vert \theta =P_{j3}^{*}\vert \theta - P_{j4}^{*}\vert \theta \\&P\left( x=5 \right) \left| \theta = P_{j4}^{*} \right| \theta -0 \end{aligned}$$

For the GRM analyses, item parameters were estimated (marginal maximum likelihood) with *mirt* (Chalmers, 2012) using default options. The graded response model can be thought of as a logistic (vs. normal ogive) re-parameterization of the item level factor model (i.e., a factor analysis based on tetrachoric correlations where item loadings and thresholds are estimated). (See Takane and de Leeuw, 1987, for specific transformation equations based on the normal ogive.)

We display the factor loadings output from *mirt* in Table 3 under the column heading lambda ($\lambda );$ these are especially high values but typical of what is found with PROMIS depression items (e.g., see Stover et al., 2019) calibrated on community samples. In Table 3, the average slope ($\alpha _{j}$ parameter) is 4.15 (*sd* $=$ 0.44). All items are highly discriminating, especially #8, “*I felt hopeless*,” with a slope of nearly 5. Indeed, the factor loading suggests that 90% (.95$^{\mathrm {2}})$ of the variance on the latent variable can be explained by responses to this one item. The average item location parameters were − 0.30, 0.32, 1.01, and 1.68 for TRC 1…4, respectively. These are not symmetrically arranged around the mean as one might expect with a fully continuous construct. Notice also that there is no location parameter lower than − 0.65, which means that no category options can differentiate among lower trait individuals, an indirect sign of a unipolar, trait.

**Table 3:** Graded response model (GRM) item parameter estimates

	Loading	GRM slope	GRM locations			
	$\lambda$	$\alpha$	$\beta _{1}$	$\beta _{2}$	$\beta _{3}$	$\beta _{4}$
*Slope and location parameterization*						
1	.92	3.86	- 0.05	.54	1.14	1.75
2	.92	4.04	- 0.19	.38	1.02	1.6
3	.93	4.22	- 0.24	.36	1.01	1.71
4	.90	3.59	- 0.65	.12	.96	1.71
5	.94	4.70	- 0.14	.40	1.03	1.61
6	.92	4.06	- 0.39	.24	.93	1.65
7	.91	3.77	- 0.62	.13	.96	1.70
8	.95	4.99	- 0.12	.41	1.01	1.69
Mean		4.15	- 0.30	.32	1.01	1.68
SD		.44	.21	.14	.06	.05

		GRM slope	GRM intercepts			
		$\alpha$	$\gamma _{1}$	$\gamma _{2}$	$\gamma _{3}$	$\gamma _{4}$
*Slope and intercept parameterization*						
1		3.86	0.19	- 2.08	- 4.39	- 6.75
2		4.04	0.76	- 1.54	- 4.13	- 6.46
3		4.22	1.02	- 1.52	- 4.28	- 7.24
4		3.59	2.34	- 0.42	- 3.44	- 6.15
5		4.70	.67	- 1.9	- 4.84	- 7.58
6		4.06	1.58	- 0.96	- 3.76	- 6.71
7		3.77	2.34	- 0.51	- 3.61	- 6.42
8		4.99	0.59	- 2.05	- 5.03	- 8.42
Mean		4.15	1.19	- 1.37	- 4.19	- 6.97
SD		.44	.76	.62	.53	.70

Finally, it is important to note that an intercept parameter $\gamma _{i}$ can be defined such that:

3 $$\begin{aligned} \gamma _{j}=-\alpha _{j}\beta _{jk} \end{aligned}$$

The intercept, shown in the bottom panel of Table 3, is the log-odds of responding above a given between category boundary when the latent trait $\theta =$ 0; the scale for the intercept is similar to a *z*-score metric where more negative values reflect categories that are more difficult to endorse, and more positive intercepts reflect categories that are relatively easier to endorse.

### LL IRT Model

The LL model was one of several possible alternative models proposed in Lucke (2013; 2015). Lucke questioned the appropriateness of using standard IRT models with addiction symptom measures, such as gambling or alcohol use. For these constructs, when data are calibrated in community samples, the base rates of behavior are very low, and the total scores tend to be highly skewed. Importantly, using a metric where the mean is the anchor for relative standing does not appear to make much sense. Rather, these types of constructs are unipolar where the low end is the absence of symptoms, not low trait standing. For the study of addictions, Lucke (2015, p. 272) writes:

The assumption of bipolarity, however, creates several problems for measuring levels of an addictive disorder. The first is that the assumption entrains trait scores that are not interpretable as a level of disorder. While it makes sense to assert that a person has a below-average ability in music or a right-of-center attitude toward gun control, it makes little or no sense to assert that a person has a below-average level of addiction to alcohol or an above-average level of addiction to gambling.

Moreover, in reference to the continuous metric in standard IRT models that range from negative to positive infinity:

The second problem is the appropriate score $\theta$* for “no disorder.” As there is no trait level less than that of “no disorder,” the anchor should therefore be located at the least possible value for the trait. Under the assumption of bipolarity, we must have $\theta$* $= \quad -\infty$. If, in addition, the trait is assumed to be a random variable following a probability density on the entire real line, then the value $\theta$* must have probability density zero. The model thus formally assumes that there are no persons without a disorder. (p. 273)

Finally, in terms of having a model that is consistent with the substantive phenomena a researcher wishes to assess:

The third and perhaps most important problem with bipolar traits is that they do not realistically represent an addictive disorder. Theories of addiction claim that “[an addictive disorder] can be usefully viewed as a behavioral manifestation of a chronic condition of the motivational system in which a reward-seeking behavior has become out of control” (West, 2006, p. 174). The excessive behavior is hypothesized to be caused by *cumulative ampliative effects *of an underlying reward system, itself arising from a nexus of personal dispositions and social influences, that are *inadequately damped *by eroded motivational constraints and ineffective social norms (Orford, 2001). Addiction is a *unipolar *disorder. Conceptualizing the level of addictive disorder as a latent trait and modeling the cumulative ampliative and dampening effects as infinitesimal multiplicative processes, Gibrat’s “law of proportional effects” implies that the trait should asymptotically follow a lognormal density (Johnson, Kotz, & Balakrishnan, 1994). (p. 273)

In the present context, we consider depression, like addictions, to be unipolar, as such, a candidate to be represented by a model with zero as an anchor. Lucke’s substantive theory of addiction as described, and assumed latent distribution, however, does not necessarily generalize to depression or any other psychopathology construct. (To read more on this, see Tomitaka et al., 2019, and Tomitaka, Kawasaki, & Furukawa, 2015, for their writings on latent distributions in distress and depression, respectively.) We return to theoretical issues later.

The polytomous version of the LL model can be written as:

4 $$\begin{aligned} P_{jk}^{*}\vert \theta = \frac{\varepsilon _{jk}\theta ^{\alpha _{j}}}{1+\varepsilon _{jk}\theta ^{\alpha _{j}}} \end{aligned}$$

and in the GRM,

$$\begin{aligned}&P(x=0)\vert \theta =1- P_{j1}^{*}\vert \theta \quad P(x=1)\vert \theta =P_{j1}^{*}\vert \theta - P_{j2}^{*}\vert \theta \\&P(x=2)\vert \theta =P_{j2}^{*}\vert \theta - P_{j3}^{*}\vert \theta \quad P(x=3)\vert \theta =P_{j3}^{*}\vert \theta - P_{j4}^{*}\vert \theta \\&P\left( x=5 \right) \left| \theta = P_{j4}^{*} \right| \theta -0 \end{aligned}$$

On the low end, theta is anchored at 0, and the high end is positive infinity. The $\varepsilon _{j}$ parameter is an item’s “easiness” (higher values are associated with item categories with higher endorsement rates, and lower values are associated with item categories with lower endorsement rates). Item slope parameters are defined exactly the same in the LL model as in the GRM. Importantly, the latent trait is now assumed log-normal; this is critically important in relating the GRM to the LL in the present evaluation.

There is no readily available software to estimate the polytomous version of the log-logistic model.^4^ For the present demonstration, however, we can take advantage of the relationship between the GRM and LL models. Specifically, we make the reasonable assumption that the slopes of the item response curves are the same in the two models. The other parameters are exponential transformations of each other. In Eq. 5, we state that trait level estimates in the LL model are the exponent of the trait level estimates in the GRM. Likewise, the easiness ($\varepsilon _{LL})$ and severity ($\delta _{LL})$ parameters in the LL model are the exponent of the intercept and location parameter in the GRM.

5 $$\begin{aligned} \theta _{LL}=\mathrm {exp}(\theta _{GRM}), \varepsilon _{LL}=\mathrm {exp}(\gamma _{GRM}), \delta _{LL}=\exp \left( \beta _{GRM} \right) \end{aligned}$$

Using these transformations, item easiness values are shown in the last four columns of Table 4. The averages are 4.43, 0.31, 0.017, and 0.0011, respectively. The item slope or discrimination values are the same as the slopes in the GRM. Finally, an item severity ($\delta _{j})$ can be defined as the point on the latent trait scale where the probability of endorsing is .50. The higher the severity, the rarer the symptom. Severities in the LL model are analogous to the locations in the GRM.

**Table 4:** Log-logistic (LL) item parameter estimates

	LL Slope	LL Easiness			
	$\alpha$	$\varepsilon _{1}$	$\varepsilon _{2}$	$\varepsilon _{3}$	$\varepsilon _{4}$
1	3.86	1.20	.124	.012	.001
2	4.03	2.14	.215	.016	.002
3	4.22	2.76	.218	.014	.001
4	3.59	10.37	.659	.032	.002
5	4.70	1.94	.150	.008	.001
6	4.05	4.85	.383	.023	.001
7	3.77	10.42	.603	.027	.002
8	4.99	1.81	.129	.007	.000
Mean	4.15	4.43	.31	.017	.0011
SD	.44	3.58	.20	.009	.0006

		LL Severities			
	$\delta _{1}$	$\delta _{2}$	$\delta _{3}$	$\delta _{4}$	
1	.95	1.72	3.11	5.73	
2	.83	1.46	2.78	4.95	
3	.79	1.43	2.75	5.56	
4	.52	1.12	2.60	5.53	
5	.87	1.50	2.80	5.02	
6	.68	1.27	2.53	5.23	
7	.54	1.14	2.61	5.48	
8	.89	1.51	2.74	5.40	
Mean	.76	1.39	2.74	5.36	
SD	.15	.19	.17	.26	

6 $$\begin{aligned} \delta _{jk}= \left( \frac{1}{\varepsilon _{jk}} \right) ^{1/\alpha _{j}} \end{aligned}$$

In the present data, the mean item severity values were 0.76, 1.39, 2.74, and 5.36, respectively. The fact that the GRM and LL models are related by an exponential transformation suggests a major model difference—individual differences at the low end of the scale are compressed and expanded at the higher end, as we will show below.

In the following section, we first provide some support for the notion that the LL model can be considered a transform of the GRM. We then explain how the models are similar or different in terms of item response curve, latent trait score estimates, and measurement precision.

### Equivalence: Some Supporting Data

We have argued that the GRM (with normality, 0, 1) and the LL (with log-normal, 0, 1) are simply transforms of each other.

Since

$$\begin{aligned} \theta _{LL}= & {} \mathrm {exp}(\theta _{GRM}) \quad \varepsilon _{LL}=\exp \left( \gamma _{GRM} \right) =\exp \left( -\alpha _{j}\beta _{jk} \right) \\&P_{jk}^{*}\vert \theta = \frac{\varepsilon _{jk}\theta ^{\alpha _{j}}}{1+\varepsilon _{jk}\theta ^{\alpha _{j}}} \quad (\mathrm{see}\,\mathrm{Equation}~4)\\&\quad = \frac{\exp \left( -\alpha _{j}\beta _{jk} \right) {\mathrm {exp}(\theta _{GRM})}^{\alpha _{j}}}{1+\exp \left( -\alpha _{j}\beta _{jk} \right) {\mathrm {exp}(\theta _{GRM})}^{\alpha _{j}}}\\&\quad = \frac{\mathrm {1}}{1+1/[\exp \left( -\alpha _{j}\beta _{jk} \right) \exp \left( \theta _{GRM} \right) ^{\alpha _{j}}]}\\&\quad = \frac{\mathrm {1}}{1+\exp \left( {-\alpha }_{j}\left( \theta _{GRM}-\beta _{jk} \right) \right) }\quad (\mathrm{see}\,\mathrm{Equation}~2) \end{aligned}$$

To provide evidence of this, we now show that the two sets of parameters imply the same response proportions and correlation matrix. In the top panel in Table 5, we display the response proportions in each category based on the 3000 subjects (these same values are reported in Table 1). In the middle column are the reproduced category proportions based on simulating 10,000 response patterns using the estimated GRM model parameters (Table 3) as true. For each simulated response pattern, we randomly sampled a trait level estimate based on the estimated Expected a Posteriori (EAP; Bock & Mislevy, 1982) trait level estimates in the sample. These GRM item parameters do an outstanding job recovering the original data, but the Chi-squares for Items #4 and #7 were significant, alpha $=$ .01. Note, however, that with $N \quad =$ 10,000 simulated response patterns, these tests are very powerful. In the bottom panel are analogous reproduced category proportions based on simulating 10,000 response patterns using the parameters of the LL model (Table 4). Again, the recovery is outstanding, and as before, Items #4 and #7 are significant (alpha $=$ .01). Finally, we note that the differences between the polychoric correlation matrices estimated within each simulated data set averaged − 0.001; stated differently, this is an empirical demonstration that the GRM and the LL models imply the same correlation matrix.

**Table 5:** Observed and model reproduced response proportions for graded response and log-logistic models

	Response proportions					
Item	0	1	2	3	4	
*Original data*						
1	.47	.21	.18	.09	.05	
2	.42	.20	.21	.11	.06	
3	.40	.21	.22	.12	.05	
4	.28	.25	.28	.14	.06	
5	.43	.20	.21	.10	.06	
6	.35	.22	.24	.14	.06	
7	.28	.25	.28	.13	.06	
8	.44	.19	.20	.12	.05	

		1	2	3	4	$\chi ^{2}$
*Reproduced from GRM*						
1	.47	.21	.18	.09	.05	2.62
2	.42	.20	.21	.11	.06	1.69
3	.40	.21	.22	.12	.05	2.63
4	.29	.24	.28	.14	.05	20.70*
5	.43	.20	.21	.10	.05	3.17
6	.36	.21	.24	.13	.06	10.84
7	.30	.24	.28	.14	.05	18.71*
8	.45	.19	.20	.12	.04	6.29

		1	2	3	4	$\chi ^{2}$
*Reproduced from LL*						
1	.47	.21	.18	.09	.04	5.92
2	.42	.20	.21	.10	.06	6.81
3	.41	.21	.22	.12	.05	5.67
4	.29	.24	.28	.14	.05	19.86*
5	.44	.20	.21	.11	.05	6.78
6	.36	.21	.24	.14	.05	8.01
7	.30	.24	.28	.13	.05	20.01*
8	.44	.19	.20	.11	.05	2.23

$^{*}$ Chi-square significant alpha $= .99$.

## Model Comparisons

### Item Response Curves

**Fig. 2:**
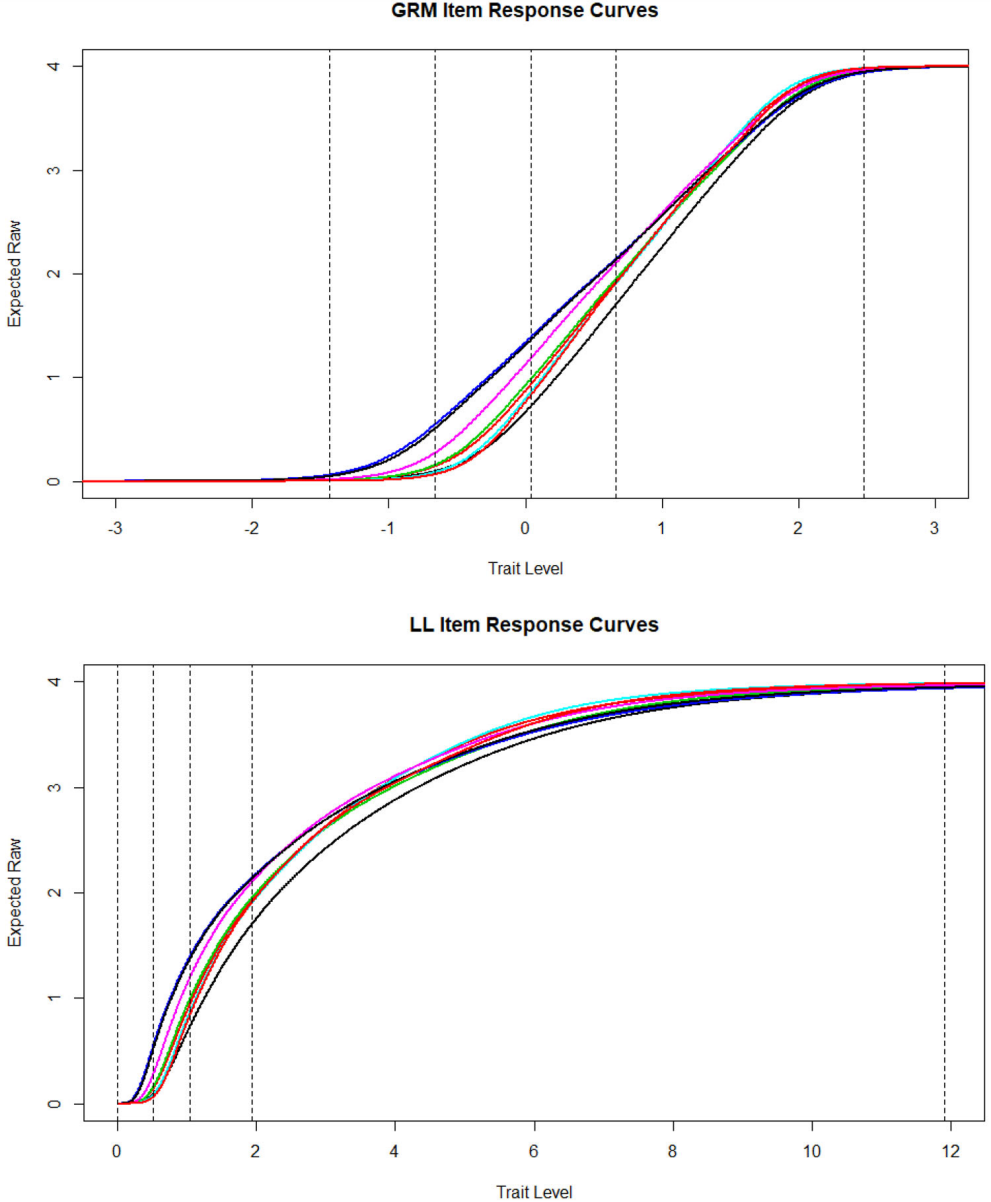
Item response curves under the graded response model and log-logistic model.

In the top panel of Fig. 2 are shown all 8 item response curves under the GRM where the x-axis ranges from − 3 to $+$ 3. The vertical lines are the minimum (− 1.43), 1st (− 0.66), 2nd (0.4) and 3rd (0.66) quartile, and max (2.47) estimated trait levels. The item response curves are logistic ogives, and they increase sharply in the trait range 0 to 2 theta, which is where the item locations are concentrated. In the bottom panel are the item response curves in the LL model. The vertical lines are the minimum (.00), 1st (.52), 2nd (1.4) and 3rd (1.94) quartile, and max (11.90). From 0 to the median trait level, the expected response item score ranges from zero to approximately 1. Likewise, from the median to the 3rd quartile, the expected score ranges from 1 to 2. Finally, the 4th quartile covers a wide range of trait scores where the expected item score ranges from approximately 2 to 4. The item response curves flatten out from theta equals roughly 8 and beyond. Comparing the upper panel with the lower, it is clear that a major difference between these models is that in the GRM, the quartiles are roughly equally spaced across the continuum, but in the LL, the first three quartiles are compressed, whereas the fourth is greatly expanded. In other words, people at higher levels of depression are more spread out in the LL, whereas at lower levels, they are compressed.

**Fig. 3:**
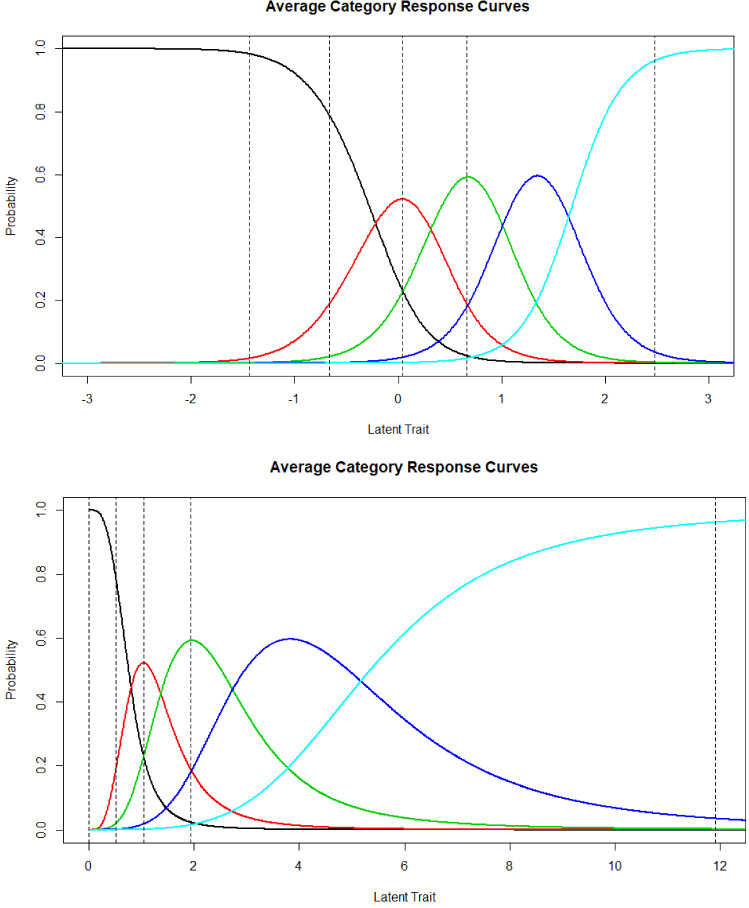
Average category response curves under the graded response model and log-logistic model.

To further illustrate the item response curves in the two models, in Fig. 3 are the average category response curves for the GRM (top panel) and the LL (bottom panel), respectively. The lines are the minimum and maximum scores, and the 1st, 2nd, and 3rd quartiles. In the GRM, a response of 1 is most likely from − infinity to approximately − 0.5 theta, a response of 1 is most likely within about a half theta unit around the mean, a response of 2 from approximately 0.5 to 1.0 theta, a response of three from 1 to 1.75 theta, and a response of 4 is most likely beyond that point. In the LL, response probabilities for the lowest two categories are squeezed very tightly from approximately 0 to 1 theta, where about 50% of respondents are located. A response of 2 is most likely from theta 1.5 to 2.5, a 3 response is most likely from 2.5 to 3 theta, a response of 4 from 3 to 5 theta; beyond that a response of 4 is most likely.

### Latent Trait Scores

**Fig. 4:**
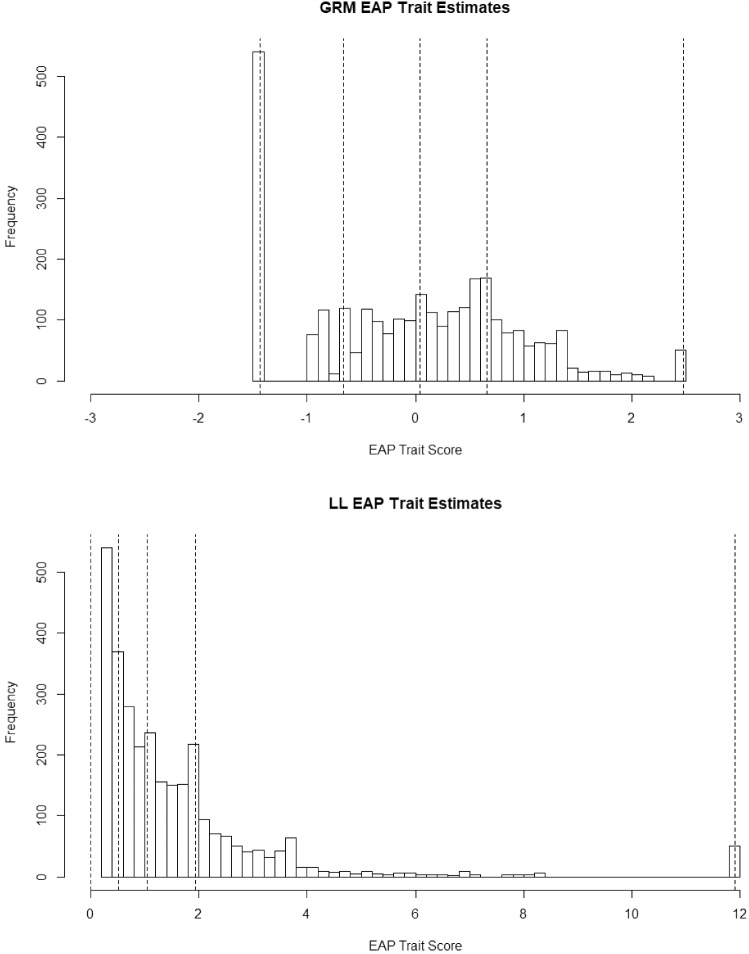
EAP trait level estimates under the graded response model and log-logistic model.

Our next set of model contrasts pertain to latent trait scores. In the top panel of Fig. 4 are the EAP trait level estimates in the GRM. The dotted lines are the minimum and maximum scores and the 1st, 2nd, and 3rd, quantiles. This is an odd distribution in that no one can score lower than − 1.65 (the minimum score) because there are no items with information in that range or that can differentiate among people at the low end. In the bottom panel, for the LL model, the distribution of trait level estimates (mean of posterior distribution) appears to be a highly positively skewed distribution. The trait level estimates in the LL were correlated .81 with the GRM estimates. In comparing the two distributions, the LL model expands the differences between high trait people in the GRM and contracts the difference between scores in the low and middle range in the GRM. This is expected because, as noted, trait level estimates in the LL model are essentially an exponential function of trait level estimates in the GRM (with the caveat that all zero response patterns are set to 0 in the LL).

**Fig. 5:**
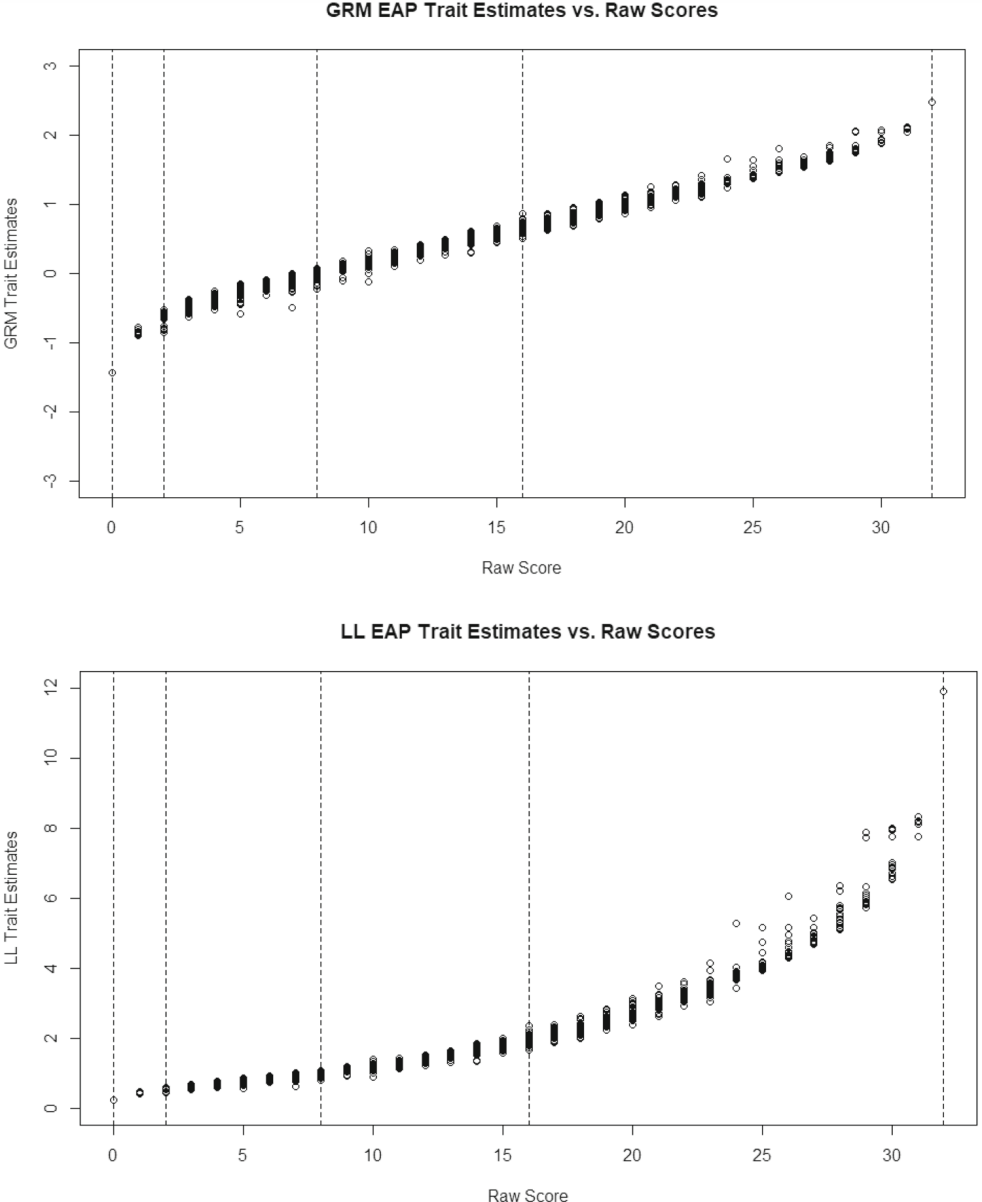
EAP trait level estimates versus raw scores under the graded response model and log-logistic.

To demonstrate model differences in scoring in a slightly different way, in the top panel of Fig. 5 is the relationship between raw scores and theta estimates in the GRM. For each 1-unit change in raw scores, the average theta estimate increases almost linearly. In the bottom portion is the relationship between raw scores and theta estimates for the LL model. Here it is clear that increases in raw scores at the low end of the scale do not change the average theta estimate much at all, but as symptoms increase, the average theta increases more and more. In short, endorsing more symptoms implies ever-increasing trait severity; symptoms have a greater effect on theta as the number of symptoms increases.

**Fig. 6:**
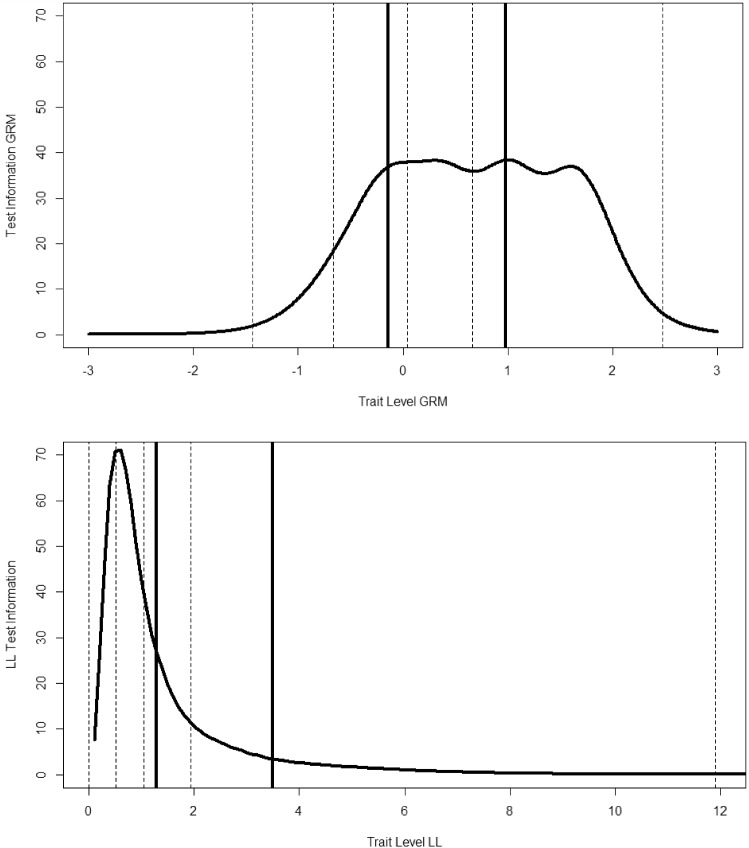
Test information under the graded response model and log-logistic model.

**Fig. 7:**
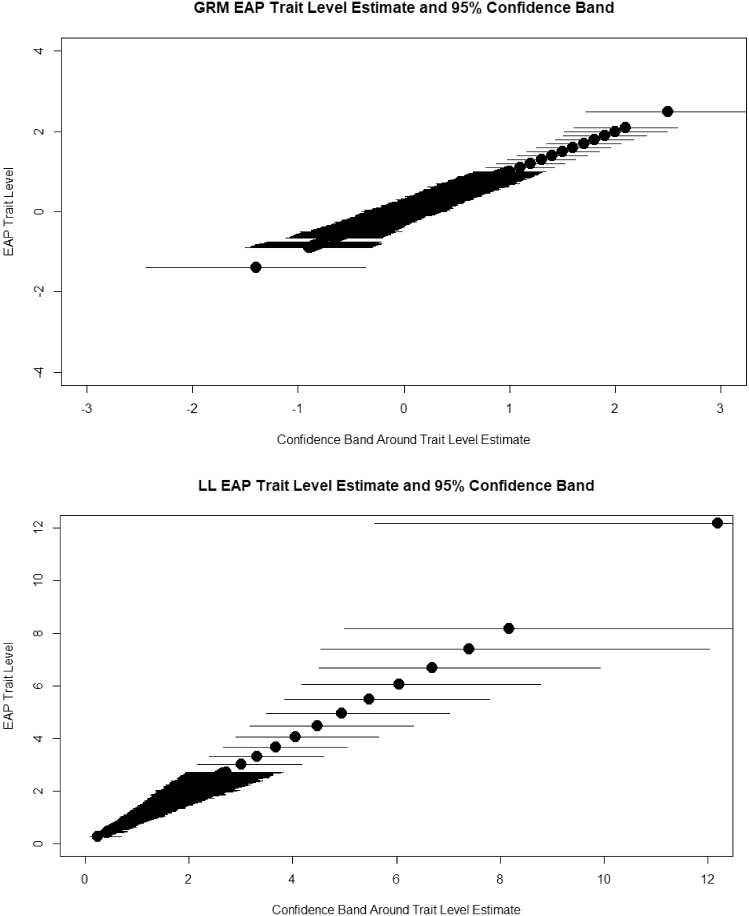
Confidence bands for trait level estimates under the graded response model and log-logistic model.

In Fig. 6, we display the test information curves for GRM and LL,^5^ respectively. The solid lines are the means for people who rated their present behavior to be limited by depression. The dotted lines are the min, 1st, 2nd, and 3rd quartile, and max scores. This figure more dramatically illustrates a major difference between the models; when the lower end is compressed, information becomes very large at the low end in the LL. Although high scores are more spread out in the LL, the information is relatively low, suggesting that although we can differentiate those who are severe versus those who are not, it is difficult to differentiate among the more severely depressed individuals. Theoretical test information functions can be useful, but we prefer to examine the trait level estimates and confidence bands. Accordingly, in Fig. 7, we show the 95% (posterior means plus and minus 2 posterior standard errors) credible bands around the EAP trait level estimates (i.e., posterior means) in the GRM. In the bottom panel of Fig. 6 are shown the estimated thetas in the LL model, as well as the estimated confidence bands. Clearly, if a person scores relatively high, we would have confidence that they were not zero or low, but almost no confidence that someone with a theta of 6 (CI approximately from 4 to 9) is reliably different than a person with theta $=$ 8 (CI approximately from 5 to above 12). To summarize, the GRM shows that the test can differentiate among people from around the mean to two theta units above the mean, whereas the LL model shows that you can differentiate between cases from non-cases but not among very severe cases.

## Discussion

The parameters of the GRM and the LL model are nonlinear transformations of each other,^6^ and although they imply response propensities that are equal and produce correlation matrices that are equivalent, they can lead to very different interpretations of the psychometric properties of a measure and the scaling of individual differences. For addictions measurement, Lucke (2013; 2015) proposed that the LL model may be useful because addiction constructs, such as alcohol abuse, yield symptom data that are highly skewed and unipolar, and thus, the goal of measurement is to scale people on a severity continuum, with no pathology at the bottom anchoring the scale. Moreover, he linked the LL model and the expectation of a log-normal distribution, to a substantive theory of addictions. In the present report, we applied both the GRM and the LL model to a self-report measure of depression. Although we do not conclude that one model has “a better fit” or is more “valid” than the other, we do suggest that the LL model may be more consistent with the construct of depression as a unipolar quasi-continuous phenomenon. Specifically, the LL model addressed our concerns with the unipolar nature of depression and the highly skewed trait distribution assumed to underlie depression.^7^ In the following, we comment on: (a) noteworthy differences between the GRM and the LL model, (b) alternative measurement models and the potential value of substantive theory in guiding measurement model selection.

### Basic Differences Between the GRM and the LL Model

Revealed in the present data are two striking differences between the GRM and LL model, and they can be expected to translate to a variety of measurement contexts where the construct under study is unipolar and the distribution of the trait is highly skewed. The first is that the two models give a very different picture of the psychometric properties of the test because the parameters in the two models are in different scales. In the GRM, location parameters (and, thus, item information) are concentrated between the mean and one standard deviation above the mean on the latent trait scale. This is considered a “peaked” test that provides information and, thus, measurement precision, for people slightly above the mean on the construct. If this were an IRT application under review for publication, the authors might have been given the all too common recommendation that, “Items need to be written to provide information in lower trait ranges.” They should challenge that request, however, and reply, “Is that possible with this depression construct, or is it essentially a unipolar-trait and meaningful only at one end?” In contrast, in the LL model, severity parameters are located mostly in the lower end of the scale. Thus, it appears that the measure basically provides a separation of individuals into cases and non-cases where individual differences within more severe trait ranges are hard to reliably distinguish, but the reliable distinction between low and high is very good. The measure can show that a person is not depressed with great precision, but making distinctions among those who are more severely depressed is challenging. In fact, it might be argued that more items need to be written for the extreme upper range of the trait.

A second difference is in the scaling of individual differences. In the GRM (and similar models), changes on the latent trait are considered important and meaningful across the trait range because the trait is a complete continuum. As test scores (symptom counts) increase, trait level estimates rise steadily as a function of symptom severity. In the LL model, because it adapts to unipolar traits, symptom variation at the low end is compressed; endorsing a few symptoms does not raise scores substantially. At the higher end, however, each additional symptom increases theta much more substantially. Is one model “better” for scaling depression? Is the relationship between latent disease state and symptom severity linear, or is it exponential? At present, we cannot answer these questions (again, see footnote 6).

We can say, however, that because the two models imply basically the same item response matrix, the conventional fit index contest would not give us these answers. In the present case, the GRM and the LL model imply the same item response data, as demonstrated, and thus, we cannot perform a nested model fit comparison. Even if we could, caution in interpretation would be advisable as one model may have more “fitting propensity” than another (Preacher, 2006; Bonifay & Cai, 2017). Consider the case of the bifactor model, which routinely “fits better” than alternatives such as the second order. Recent psychometric work has suggested that such “better fit” may have more do to with biases in model comparison than actual superiority or verisimilitude of the bifactor (Forbes et al., 2021; Greene et al., 2019; Markon, 2019). Moreover, Reise et al. (2016) suggest that the bifactor may sometimes “fit better” merely because it better models faulty response patterns. If statistics cannot provide definitive answers to model choice, then how can one choose between models? We consider that question next.

### Alternative Models, Substantive Theory, and IRT

We have contrasted two possible measurement models for depression and highlighted similarities and differences. The two models presented here are but two of many possibilities. Indeed, Lucke (2013; 2015) introduced and applied three possible models for the item response curve, LL, log-normal, and Weibull. These models can be considered as part of a much wider class of generalized linear models that Mellenbergh (1994) described. Alternatives to the standard logistic graded response model are not new. Many years ago, for example, Goldstein (1980) questioned the appropriateness of routinely applying logistic IRT models given that many functions provide monotonically increasing item response curves, and they would be expected to yield somewhat different scalings of individual differences.

Nevertheless, standard logistic IRT models have been both thoughtfully and reflexively applied across a wide variety of construct domains, as illustrated by the quotations below: van der Mass et al. (2011, p. 353): “In introductions to IRT, the preference for the logistic equation is typically explained in terms of statistical or measurement theoretical convenience. However, from a substantive point of view, the lack of a psychological justification for this key property of the measurement model compromises test validity. The reason is that validity requires a causal mechanism linking the trait or ability with the item responses (Borsboom & Mellenbergh, 2007; Borsboom et al., 2004).”

Garcia-Perez (1999, p. 75): “No one seems to have questioned whether, in the real world, logistic item and examinee parameters are actually there to be recovered or, in other words, whether the mathematical form of the IRF, can be derived from a psychological theory of performance in objective tests as opposed to adopting a convenient function that the data are forced to fit by fiat.”

Finally, (p. 91), “According to Gulliksen (1961, p. 101), psychometric models should establish “the relation between ability of the individual and his observed score on the test” But establishing that relationship implies an exercise in substantive theory and model building before any function is fitted to the data.”

How can a shift to linking substantive theory to measurement model form in IRT occur? One clear example is Lucke’s (2013) justification of a log-logistic form for addiction measurement based on specific addiction theories. One does not have to agree with his theory to acknowledge his effort to link the scaling of individual differences to a theory of addiction.

Ultimately, evidence for validity of a particular scaling must come from outside the test. Given the impressive, recent advances in neuropsychology and the measurement of brain functioning, structural models, such as Multiple Indicator Multiple Cause models, that can integrate these advances within commonly used structural equation models (e.g., Kievit et al., 2011a; b), as well as the call to reconceptualize and flesh out the biological and cognitive mechanisms and processes involved in important pathology constructs in Research Domain Criteria (Insel, 2010), this is an excellent time to begin integrating psychological theory into psychological measurement, in general, and IRT modeling, in particular. We believe that this is an important topic for future psychometric work, for promoting psychologically and biologically informed measurement models can lead to a more valid scaling of individual differences which, in turn, can improve the validity of inferences drawn from psychological research. We do so, however, with a good understanding of the many challenges and the required partnerships.

## Supplementary Information

Below is the link to the electronic supplementary material.

Supplementary material 1 (pdf 231 KB)

## Footnotes

1 To simplify our presentation, we will sidestep the confusing distinctions between the terms, HRQoL and PRO by using the term PRO from this point on. Our running example is a depression measure, which is commonly considered a PRO. Much of what we write about PRO measures, however, also applies to HRQoL measures.

2 The results of zero-inflated models are somewhat similar to what occurs if all zero scores were to be eliminated, and the model is estimated on the remaining data. Due to eliminating the zeros, the sample becomes more homogeneous and slope parameters are expected to decrease.

3 We recognize that one cannot be certain that the observed score distribution reflects the latent trait distribution. That said, to the extent that these raw scores do reflect the “true” distribution, we know of no IRT approaches for addressing non-normal distributions that can correct for a half-normal distribution.

4 Magnus and Liu (2018) developed a maximum likelihood method for estimating a zero-inflated version of the polytomous log-logistic model based on R code; however, even if we were to adapt their code for our purposes, there still is no marginal maximum likelihood estimation method readily available that would be needed for a fair comparison with the estimated parameters for the GRM. Regardless, the purpose of the present article is largely didactic, and we do not wish to conflate estimation issues (the routine’s accuracy and which priors to use) with the messages we are trying to convey.

5 We thank Maxwell Mansolf for writing the R code to estimate information in the LL model.

6 To be clear, we used these theoretical transformations to demonstrate a psychometric point. In practice, Bayesian or marginal maximum likelihood estimation routines should be developed and evaluated.

7 Clearly, if one could show that scores based on the LL model were more strongly related to, say, mental health care utilization, suicide attempts, and so forth, this would provide support for using it in applied settings.
