# Supplementary material for: Matching IRT Models to Patient-Reported Outcomes Constructs: The Graded Response and Log-Logistic Models for Scaling Depression
Source: Psychometrika. 2021 Aug 31;86(3):800–24. doi: 10.1007/s11336-021-09802-0 (PMC8437930; doi:10.1007/s11336-021-09802-0)
Supplement: Supplementary file 1 — Supplementary material 1 (pdf 231 KB) [file 11336_2021_9802_MOESM1_ESM.pdf]

Matching IRT Models to PRO Constructs: The Graded Response and Log-Logistic Models for Scaling Depression

```
# load data and required packages
setwd(dirname(rstudioapi::getActiveDocumentContext()$path))
load("Recentercompletes.rdata")
library(psych)
library(mirt)
```

Depression diagnosis frequencies:

```
#Qclinic01a {Yes} Have you ever been told by a doctor or a health professional
# that you had any of the following?
DepDiag <- table$Qclinic01a_15 # depression
DepDiag <- as.data.frame(DepDiag)
table(DepDiag)
```

```
## DepDiag
##    0    1
## 2267  732
```

Current depression frequencies:

```
# Qclinic02a15  {Depression} Are any of your current activities limited by the following conditions?
CurrentDep <- table$Qclinic02a15
CurrentDep <- as.data.frame(CurrentDep)
table(CurrentDep)
```

```
## CurrentDep
##   0   1
## 307 396
```

From the original dataset, we created a subset of the data with only the depression items and saved it as an object called `Depress`.

```
# these are the depression items in the dataset
# QEDDEP04 QEDDEP05 QEDDEP06 QEDDEP17 QEDDEP22 QEDDEP29 QEDDEP36 QEDDEP41
Depress <- matrix(0,3000,8)

Depress[,1] <- table$QEDDEP04
Depress[,2] <- table$QEDDEP05
Depress[,3] <- table$QEDDEP06
Depress[,4] <- table$QEDDEP17
Depress[,5] <- table$QEDDEP22
Depress[,6] <- table$QEDDEP29
Depress[,7] <- table$QEDDEP36
Depress[,8] <- table$QEDDEP41

# Converting to a 0 to 4 scale
Depress <- as.data.frame(Depress)
Depress <- Depress - 1
```

Code for analyses displayed in Tables 2 - 5:

Table 2.

The code and results for Table 2. which computes and displays classical test theory results shown in Table 2.

```
# compute descriptive statistics and classical test theory results
Dalpha <- alpha(Depress)
Dalpha
```

```
##
## Reliability analysis
## Call: alpha(x = Depress)
##
##   raw_alpha std.alpha G6(smc) average_r S/N    ase mean  sd median_r
##       0.96      0.96    0.96      0.75  24 0.0011  1.2 1.1     0.75
##
##  lower alpha upper     95% confidence boundaries
## 0.96 0.96 0.96
##
##  Reliability if an item is dropped:
##    raw_alpha std.alpha G6(smc) average_r S/N alpha se   var.r med.r
## V1      0.96      0.96    0.95      0.76  22   0.0012 0.00068  0.75
## V2      0.96      0.96    0.95      0.75  21   0.0012 0.00088  0.75
## V3      0.95      0.95    0.95      0.75  21   0.0013 0.00095  0.75
## V4      0.96      0.96    0.95      0.76  22   0.0012 0.00070  0.75
## V5      0.95      0.95    0.95      0.75  21   0.0013 0.00081  0.74
## V6      0.95      0.95    0.95      0.75  21   0.0013 0.00100  0.74
## V7      0.96      0.96    0.95      0.76  22   0.0012 0.00085  0.75
## V8      0.95      0.95    0.95      0.75  21   0.0013 0.00078  0.74
##
##  Item statistics
##       n raw.r std.r r.cor r.drop mean  sd
## V1 3000  0.87  0.87  0.84   0.83  1.0 1.2
## V2 3000  0.88  0.88  0.86   0.84  1.2 1.3
## V3 3000  0.89  0.89  0.87   0.85  1.2 1.2
## V4 3000  0.87  0.87  0.85   0.83  1.4 1.2
## V5 3000  0.90  0.90  0.88   0.87  1.2 1.2
## V6 3000  0.89  0.89  0.87   0.85  1.3 1.2
## V7 3000  0.88  0.88  0.86   0.84  1.4 1.2
## V8 3000  0.90  0.90  0.89   0.87  1.1 1.2
##
## Non missing response frequency for each item
##       0    1    2    3    4 miss
## V1 0.47 0.21 0.18 0.09 0.05    0
## V2 0.42 0.20 0.21 0.11 0.06    0
## V3 0.40 0.21 0.22 0.12 0.05    0
## V4 0.28 0.25 0.28 0.14 0.06    0
## V5 0.43 0.20 0.21 0.10 0.06    0
## V6 0.35 0.22 0.24 0.14 0.06    0
## V7 0.28 0.25 0.28 0.13 0.06    0
## V8 0.44 0.19 0.20 0.12 0.05    0
```

Table 3.

The following code applies a one-factor GRM and creates an object called `DGRMPar`.

```r
#table 3 code
DGRMPar <- mirt(Depress, 1, itemtype = 'graded')
```

The following code extracts the factor loadings from the one-factor GRM:

```r
S <- summary(DGRMPar)  # factor analysis results
S$rotF
```

```
##       F1    h2
## V1 0.915 0.837
## V2 0.921 0.849
## V3 0.928 0.860
## V4 0.904 0.817
## V5 0.940 0.884
## V6 0.922 0.850
## V7 0.912 0.831
## V8 0.947 0.896
##
## SS loadings:  6.825
## Proportion Var:  0.853
##
## Factor correlations:
##
##    F1
## F1  1
##           F1
## V1 0.9150859
## V2 0.9214837
## V3 0.9275416
## V4 0.9038205
## V5 0.9402478
## V6 0.9221017
## V7 0.9115672
## V8 0.9465380
```

The following code extracts the Slope/Location, Slope/Intercept Parameterization, and means of the parameters from the one-factor GRM:

```r
# Slope/Location Parameterization
ACcoef1 <- coef(DGRMPar, IRTpars = TRUE, as.data.frame = FALSE, simplify = TRUE)$items
round(ACcoef1,2)
```

```
##       a    b1   b2   b3   b4
## V1 3.86 -0.05 0.54 1.14 1.75
## V2 4.04 -0.19 0.38 1.02 1.60
## V3 4.22 -0.24 0.36 1.01 1.71
## V4 3.59 -0.65 0.12 0.96 1.71
## V5 4.70 -0.14 0.40 1.03 1.61
## V6 4.06 -0.39 0.24 0.93 1.65
## V7 3.77 -0.62 0.13 0.96 1.70
## V8 4.99 -0.12 0.41 1.01 1.69
```

```r
apply(ACcoef1,2,mean) # Means of Slope and Locations
```

```
##          a         b1         b2         b3         b4
##  4.1553153 -0.2998931  0.3226920  1.0062655  1.6781098
```

```r
#Slope/Intercept Parameterization
ACcoef2 <- coef(DGRMPar, IRTpars = FALSE, as.data.frame = FALSE, simplify = TRUE)$items
round(ACcoef2,2)
```

```
##      a1   d1    d2    d3    d4
## V1 3.86 0.19 -2.08 -4.39 -6.75
## V2 4.04 0.76 -1.54 -4.13 -6.46
## V3 4.22 1.02 -1.52 -4.28 -7.24
## V4 3.59 2.34 -0.42 -3.44 -6.15
## V5 4.70 0.67 -1.90 -4.84 -7.58
## V6 4.06 1.58 -0.96 -3.76 -6.71
## V7 3.77 2.34 -0.51 -3.61 -6.42
## V8 4.99 0.59 -2.05 -5.03 -8.42
```

```r
apply(ACcoef1,2,mean) # Means of Slope and Intercepts
```

```
##          a         b1         b2         b3         b4
##  4.1553153 -0.2998931  0.3226920  1.0062655  1.6781098
```

Table 4.

The following code creates log logistic transformations for the easiness parameter from the intercept parameter:

```r
# slope estimates
EtaPar <- ACcoef2[,1]
  # EtaPar

# intercepts transformed into easiness
EpsPar <- exp(ACcoef2[,2:5])
  # EpsPar

new <- cbind(EtaPar,EpsPar)
new
```

```
##      EtaPar        d1        d2          d3           d4
## V1 3.862223  1.203338 0.1243233 0.012424311 0.0011760797
## V2 4.037838  2.140417 0.2151002 0.016081368 0.0015700461
## V3 4.224220  2.765958 0.2182501 0.013873116 0.0007143841
## V4 3.594905 10.370899 0.6588765 0.032181553 0.0021417246
## V5 4.699981  1.945835 0.1495576 0.007897930 0.0005110856
## V6 4.055891  4.852201 0.3832374 0.023221298 0.0012230532
## V7 3.773521 10.424150 0.6034493 0.026935690 0.0016249615
## V8 4.993942  1.811765 0.1289405 0.006546584 0.0002196552
```

The following code computes item severities by transforming easiness estimates:

```r
# computing severity from easiness parameter
Sev <- (1/new[,2:5])^(1/new[,1])
Sev
```

```
##           d1       d2       d3       d4
## V1 0.9532047 1.715682 3.114782 5.734788
## V2 0.8282275 1.463108 2.781104 4.948263
## V3 0.7859626 1.433803 2.752981 5.556086
## V4 0.5217097 1.123062 2.601005 5.527202
## V5 0.8679363 1.498212 2.801169 5.015604
## V6 0.6774518 1.266771 2.528708 5.225221
```

```
## V7 0.5372973 1.143224 2.605974 5.484526
## V8 0.8878038 1.507086 2.737328 5.401809
```

Table 5.

The response proportions from the original data (top panel) are duplicated from Table 2.

The response proportions reproduced from the GRM (middle panel) were created by simulating 10,000 response patterns using the estimated GRM model parameters (Table 3) as true. The following code shows the simulation:

```
# simulation code here
Response <- matrix(0,10000,8)
TempR <- matrix(0,8,5)

for (i in 1:10000) {
  RTheta <- sample(DThetaIRT[,1],1, replace=TRUE)
  for (j in 1:8) {
    U <- runif(1,0,1)

    P1 <- 1 / (1 + exp(-ACcoef1[j,1] * (RTheta - ACcoef1[j,2])))
    P2 <- 1 / (1 + exp(-ACcoef1[j,1] * (RTheta - ACcoef1[j,3])))
    P3 <- 1 / (1 + exp(-ACcoef1[j,1] * (RTheta - ACcoef1[j,4])))
    P4 <- 1 / (1 + exp(-ACcoef1[j,1] * (RTheta - ACcoef1[j,5])))

    C1 <- 1 - P1
    C2 <- P1 - P2
    C3 <- P2 - P3
    C4 <- P3 - P4
    C5 <- P4 - 0

    if (U <= C1) {Response[i,j] = 0}
    if (U > C1 & U <= C1 + C2) {Response[i,j] = 1}
    if (U > C1 + C2 & U <= C1 + C2 + C3) {Response[i,j] = 2}
    if (U > C1 + C2 + C3 & U <= C1 + C2 + C3 + C4) {Response[i,j] = 3}
    if (U > C1 + C2 + C3 + C4) {Response[i,j] = 4}

      }
}

TempR[1,] <- table(Response[,1])/10000
TempR[2,] <- table(Response[,2])/10000
TempR[3,] <- table(Response[,3])/10000
TempR[4,] <- table(Response[,4])/10000
TempR[5,] <- table(Response[,5])/10000
TempR[6,] <- table(Response[,6])/10000
TempR[7,] <- table(Response[,7])/10000
TempR[8,] <- table(Response[,8])/10000

round(Dalpha$response.freq[,1:5],2)
round(TempR, 2)

library(psych)

SimDataGRM <- Response
```

```r
RGRM <- polychoric(SimDataGRM,smooth=TRUE)$rho
RGRM

# convert to frequencies
XObs <- TempR * 10000
XExp  <- Dalpha$response.freq * 10000


TempChi <- matrix(0,8,1)

for (i in 1:8) {
  for (j in 1:5) {
    TempChi[i,1] <- TempChi[i,1] + (XObs[i,j] - XExp[i,j])^2 / XExp[i,j]
  }
}
TempChi

qchisq(.95, df=4)
```

The response proportions reproduced from the LL (bottom panel) were created by simulating 10,000 response patterns using the estimated LL model parameters (Table 3) as true. The following code shows the simulation:

```r
# simulation code here

Response <- matrix(0,10000,8)
TempR <- matrix(0,8,5)

for (i in 1:10000) {
  RTheta <- sample(LLTheta,1, replace=TRUE)
  for (j in 1:8) {
    U <- runif(1,0,1)

    T1 <- EpsPar[j,1] * RTheta^EtaPar[j]
    T2 <- EpsPar[j,2] * RTheta^EtaPar[j]
    T3 <- EpsPar[j,3] * RTheta^EtaPar[j]
    T4 <- EpsPar[j,4] * RTheta^EtaPar[j]

    P1 <- T1 / (1 + T1)
    P2 <- T2 / (1 + T2)
    P3 <- T3 / (1 + T3)
    P4 <- T4 / (1 + T4)

    C1 <- 1 - P1
    C2 <- P1 - P2
    C3 <- P2 - P3
    C4 <- P3 - P4
    C5 <- P4 - 0

    if (U <= C1) {Response[i,j] = 0}
    if (U > C1 & U <= C1 + C2) {Response[i,j] = 1}
    if (U > C1 + C2 & U <= C1 + C2 + C3) {Response[i,j] = 2}
    if (U > C1 + C2 + C3 & U <= C1 + C2 + C3 + C4) {Response[i,j] = 3}
    if (U > C1 + C2 + C3 + C4) {Response[i,j] = 4}
```

```
  }
}

TempR[1,] <- table(Response[,1])/10000
TempR[2,] <- table(Response[,2])/10000
TempR[3,] <- table(Response[,3])/10000
TempR[4,] <- table(Response[,4])/10000
TempR[5,] <- table(Response[,5])/10000
TempR[6,] <- table(Response[,6])/10000
TempR[7,] <- table(Response[,7])/10000
TempR[8,] <- table(Response[,8])/10000

round(Dalpha$response.freq[,1:5],2)
round(TempR, 2)

SimDataGRM <- Response
RLL <- polychoric(SimDataGRM,smooth=TRUE)$rho
RLL

round(RLL - RGRM, 2)

# convert to frequencies
XObs <- TempR * 10000
XExp  <- Dalpha$response.freq * 10000
TempChi <- 0

for (i in 1:8) {
  for (j in 1:5) {
    TempChi <- TempChi + (XObs[i,j] - XExp[i,j])^2 / XExp[i,j]
  }
}
TempChi


TempChi <- matrix(0,8,1)

for (i in 1:8) {
  for (j in 1:5) {
    TempChi[i,1] <- TempChi[i,1] + (XObs[i,j] - XExp[i,j])^2 / XExp[i,j]
  }
}
TempChi

qchisq(.99, df=4)
```

Code for analyses shown in Figures 1-7

Figure 1

The following code generates a histogram of Depression Raw Scores. Solid vertical line indicates the mean of the raw scores. Dashed vertical lines are at one standard deviation above and below the mean.

```
temp <- seq(-0.5,40.5,1) # setting up breaks for graph

DRaw <- rowSums(Depress)  # compute raw scores

hist(DRaw, xlab = "Raw Score", breaks=temp, xlim=c(0,40), main="Unit Weighted Composite Scores")
  abline(v=mean(DRaw), lwd=2)
  abline(v=mean(DRaw)+sd(DRaw), lwd=2, lty=2)
  abline(v=mean(DRaw)-sd(DRaw), lwd=2, lty=2)
```

The following code removes cases where the depression raw score was zero:

```
# Data Without the Zeros
newdata <- DRaw[ which(DRaw > 0)]  # take out the zeros
summary(newdata)
```

```
##    Min. 1st Qu.  Median    Mean 3rd Qu.    Max.
##    1.00    5.00   12.00   12.14   17.00   32.00
```

The following code generates a histogram of Depression Raw Scores excluding all zero response patterns. Solid vertical line indicates the mean of the raw scores. Dashed vertical lines are at one standard deviation above and below the mean.

```
#### Figure 1 Bottom Panel
temp <- seq(0.5,40.5,1) # set up the histogram breaks
hist(newdata, xlab = "Raw Score", breaks=temp, xlim=c(0,40), main="Unit Weighted Composite Scores")
  abline(v=mean(newdata), lwd=2)
  abline(v=mean(newdata)+sd(newdata), lwd=2, lty=2)
  abline(v=mean(newdata)-sd(newdata), lwd=2, lty=2)
```

Figure 2

The following code generates θ scores for each participant/observation and computes the depression raw scores for each participant:

```
# generate theta scores
DThetaIRT <- fscores(DGRMPar, method = "EAP", full.scores = TRUE)
# head(DThetaIRT)
```

The following code creates the response curves for the GRM. The dashed vertical lines indicate the Min. value, 1st Quartile, Median, Mean, 3rd Quartile, Max value.

```
ExpRawGRM <- matrix(0,801,8)
# ACcoef1

CatExpGRM <- matrix(0,801,5)

ThetaGRM <- matrix(seq(-4,4,.01))
for (i in 1:8) {
  for (j in 1:801) {
    P1 <- 1 / (1 + exp(-ACcoef1[i,1] * (ThetaGRM[j] - ACcoef1[i,2])))
    P2 <- 1 / (1 + exp(-ACcoef1[i,1] * (ThetaGRM[j] - ACcoef1[i,3])))
    P3 <- 1 / (1 + exp(-ACcoef1[i,1] * (ThetaGRM[j] - ACcoef1[i,4])))
```

```
    P4 <- 1 / (1 + exp(-ACcoef1[i,1] * (ThetaGRM[j] - ACcoef1[i,5])))
    C1 <- 1 - P1
    C2 <- P1 - P2
    C3 <- P2 - P3
    C4 <- P3 - P4
    C5 <- P4 - 0
    CatExpGRM[j,1] <- CatExpGRM[j,1] + C1
    CatExpGRM[j,2] <- CatExpGRM[j,2] + C2
    CatExpGRM[j,3] <- CatExpGRM[j,3] + C3
    CatExpGRM[j,4] <- CatExpGRM[j,4] + C4
    CatExpGRM[j,5] <- CatExpGRM[j,5] + C5
    P <- 0 * C1 + 1 * C2 + 2 * C3 + 3 * C4 + 4 * C5
    ExpRawGRM[j,i] <- ExpRawGRM[j,i] + P
  }
}

# Item Response Curve
matplot(ThetaGRM, ExpRawGRM, type = 'l', lwd=c(2), xlab="Trait Level", ylab="Expected Raw",
     main="GRM Item Response Curves", ylim=c(0,4), lty=c(1), xlim=c(-3,3))
S <- summary(DThetaIRT[,1])

abline(v=S[1], lwd=1, lty=2)
abline(v=S[2], lwd=1, lty=2)
abline(v=S[3], lwd=1, lty=2)
abline(v=S[5], lwd=1, lty=2)
abline(v=S[6], lwd=1, lty=2)
```

The following code generates the response curves for the LL model. The dashed vertical lines indicate the Min. value, 1st Quartile, Median, Mean, 3rd Quartile, Max value.

```
LLTheta <- exp(DThetaIRT[,1])
for (i in 1:3000) {
  if(DRaw[i] == 0) {LLTheta[i] <- 0}
}
```

```
ExpRawLL <- matrix(0,2001,8)

CatExpLL <- matrix(0,2001,5)

ThetaLL <- matrix(seq(0,20,.01))

for (i in 1:8) {
  for (j in 1:2001) {

    T1 <- EpsPar[i,1] * ThetaLL[j]^EtaPar[i]
    T2 <- EpsPar[i,2] * ThetaLL[j]^EtaPar[i]
    T3 <- EpsPar[i,3] * ThetaLL[j]^EtaPar[i]
    T4 <- EpsPar[i,4] * ThetaLL[j]^EtaPar[i]

    P1 <- T1 / (1 + T1)
    P2 <- T2 / (1 + T2)
    P3 <- T3 / (1 + T3)
    P4 <- T4 / (1 + T4)
```

```
    C1 <- 1 - P1
    C2 <- P1 - P2
    C3 <- P2 - P3
    C4 <- P3 - P4
    C5 <- P4 - 0

    CatExpLL[j,1] <- CatExpLL[j,1] + C1
    CatExpLL[j,2] <- CatExpLL[j,2] + C2
    CatExpLL[j,3] <- CatExpLL[j,3] + C3
    CatExpLL[j,4] <- CatExpLL[j,4] + C4
    CatExpLL[j,5] <- CatExpLL[j,5] + C5

    P <- 0 * C1 + 1 * C2 + 2 * C3 + 3 * C4 + 4 * C5

    ExpRawLL[j,i] <- ExpRawLL[j,i] + P
  }
}

# Item Response Curve
matplot(ThetaLL, ExpRawLL, type = 'l', lwd=c(2), xlab="Trait Level", ylab="Expected Raw",
        main="LL Item Response Curves", ylim=c(0,4), lty=c(1), xlim=c(0,12))

S <- summary(LLTheta)

abline(v=S[1], lwd=1, lty=2)
abline(v=S[2], lwd=1, lty=2)
abline(v=S[3], lwd=1, lty=2)
abline(v=S[5], lwd=1, lty=2)
abline(v=S[6], lwd=1, lty=2)
```

Figure 3

The following code generates category response curves from the GRM. The dashed vertical lines indicate the Min. value, 1st Quartile, Median, Mean, 3rd Quartile, Max value.

```
# Category Response Curves for GRM
matplot(ThetaGRM, CatExpGRM/8, type = 'l', lwd=c(2), xlab="Latent Trait", ylab="Probability",
        main="GRM Average Category Response Curves", ylim=c(0,1), lty=c(1), xlim=c(-3,3))
S <- summary(DThetaIRT[,1])

abline(v=S[1], lwd=1, lty=2)
abline(v=S[2], lwd=1, lty=2)
abline(v=S[3], lwd=1, lty=2)
abline(v=S[5], lwd=1, lty=2)
abline(v=S[6], lwd=1, lty=2)
```

The following code generates category response curves from the LL model. The dashed vertical lines indicate the Min. value, 1st Quartile, Median, Mean, 3rd Quartile, Max value.

```
# Category Response Curves for LL
matplot(ThetaLL, CatExpLL/8, type = 'l', lwd=c(2), xlab="Latent Trait", ylab="Probability",
        main="LL Average Category Response Curves", ylim=c(0,1), lty=c(1), xlim=c(0,12))
S <- summary(LLTheta)

abline(v=S[1], lwd=1, lty=2)
```

```
abline(v=S[2], lwd=1, lty=2)
abline(v=S[3], lwd=1, lty=2)
abline(v=S[5], lwd=1, lty=2)
abline(v=S[6], lwd=1, lty=2)
```

Figure 4

The following code generates a histogram of the EAP trait level estimates in the GRM model. The dashed vertical lines indicate the Min. value, 1st Quartile, Median, Mean, 3rd Quartile, Max value.

```
hist(DThetaIRT[,1], breaks=50, xlab="EAP Trait Score", ylab="Frequency",
     main="GRM EAP Trait Estimates", xlim=c(-3,3))
S <- summary(DThetaIRT[,1])

abline(v=S[1], lwd=1, lty=2)
abline(v=S[2], lwd=1, lty=2)
abline(v=S[3], lwd=1, lty=2)
abline(v=S[5], lwd=1, lty=2)
abline(v=S[6], lwd=1, lty=2)
```

```
aggregate(DThetaIRT[,1], DepDiag, mean)
```

```
##   DepDiag          x
## 1       0 -0.2052106
## 2       1  0.6446639
```

The following code generates a histogram of the EAP trait level estimates in the LL model. The dashed vertical lines indicate the Min. value, 1st Quartile, Median, Mean, 3rd Quartile, Max value.

```
hist(LLTheta, breaks=50, xlab="EAP Trait Score", ylab="Frequency",
     main="LL EAP Trait Estimates", xlim=c(0,12))
S <- summary(LLTheta)

abline(v=S[1], lwd=1, lty=2)
abline(v=S[2], lwd=1, lty=2)
abline(v=S[3], lwd=1, lty=2)
abline(v=S[5], lwd=1, lty=2)
abline(v=S[6], lwd=1, lty=2)
```

Figure 5.

The following code generates a point plot that shows the relationship between raw scores and theta estimates in the GRM.The dashed vertical lines indicate the Min. value, 1st Quartile, Median, Mean, 3rd Quartile, Max value.

```
plot(DRaw, DThetaIRT[,1],  xlab="Raw Score", ylab="GRM Trait Estimates",
     main="GRM EAP Trait Estimates vs. Raw Scores", xlim=c(0,32), ylim=c(-3,3),
     pch=ifelse(DepDiag ==1,19,1),
     col=ifelse(DepDiag ==1,"red","black"))
S <- summary(DRaw)

abline(v=S[1], lwd=1, lty=2)
abline(v=S[2], lwd=1, lty=2)
abline(v=S[3], lwd=1, lty=2)
abline(v=S[5], lwd=1, lty=2)
abline(v=S[6], lwd=1, lty=2)
```

The following code generates a point plot that shows the relationship between raw scores and theta estimates from the LL model. The dashed vertical lines indicate the Min. value, 1st Quartile, Median, Mean, 3rd Quartile, Max value.

```
plot(DRaw, LLTheta,  xlab="Raw Score", ylab="LL Trait Estimates",
     main="LL EAP Trait Estimates vs. Raw Scores", xlim=c(0,32), ylim=c(0,12))
S <- summary(DRaw)

abline(v=S[1], lwd=1, lty=2)
abline(v=S[2], lwd=1, lty=2)
abline(v=S[3], lwd=1, lty=2)
abline(v=S[5], lwd=1, lty=2)
abline(v=S[6], lwd=1, lty=2)
```

Figure 6

The following code generates the test information curves for the GRM.The dashed vertical lines indicate the Min. value, 1st Quartile, Median, Mean, 3rd Quartile, Max value.

```
NewTheta <- seq(-3,3,.01)
T <- testinfo(DGRMPar, NewTheta)

plot(NewTheta,T, type="l", xlab="Trait Level GRM", ylab="Test Information GRM", ylim=c(0,70), lwd=4)
abline(v=-0.14, lwd=4)
abline(v=0.98, lwd=4)

S <- summary(DThetaIRT[,1])

abline(v=S[1],lty=2)
abline(v=S[2],lty=2)
abline(v=S[3],lty=2)
abline(v=S[5],lty=2)
abline(v=S[6],lty=2)
```

Note: For code for log-logistic information, inquire from authors.

Figure 7

The following code generates the 95% confidence bands around the EAP trait level estimates in the GRM.

```
DThetaIRTWSE <- fscores(DGRMPar, method = "EAP", full.scores = TRUE, full.scores.SE=TRUE)

DThetaIRTWSE[,1] <- signif(DThetaIRTWSE[,1],digits=2)
DThetaIRTWSE[,2] <- signif(DThetaIRTWSE[,2],digits=2)

aggdata <-aggregate(DThetaIRTWSE, by=list(DThetaIRTWSE[,1]),
                    FUN=mean)
# dim(aggdata)
# head(aggdata)

plot(aggdata[,1], aggdata[,1], xlim=c(-3,3), ylim=c(-4,4), type="p", pch=19, cex=2,
     xlab="Confidence Band Around Trait Level Estimate", ylab="EAP Trait Level",
     main="GRM EAP Trait Level Estimate and 95% Confidence Band")

for (i in 1:274) {
  segments(y0 = aggdata[,1], y1 = aggdata[,1], x0 = aggdata[,1],
```

```
           x1 = aggdata[,1] + 2 * aggdata[,3])
}

for (i in 1:274) {
  segments(y0 = aggdata[,1], y1 = aggdata[,1], x0 = aggdata[,1],
           x1 = aggdata[,1] - 2 * aggdata[,3])
}
```

The following code generates the 95% confidence bands around the EAP trait level estimates in the LL model.

```
plot(exp(aggdata[,1]), exp(aggdata[,1]), xlim=c(0,12), ylim=c(0,12), type="p", pch=19, cex=2,
     xlab="Confidence Band Around Trait Level Estimate", ylab="EAP Trait Level",
     main="LL EAP Trait Level Estimate and 95% Confidence Band")


for (i in 1:274) {
  segments(y0 = exp(aggdata[,1]), y1 = exp(aggdata[,1]), x0 = exp(aggdata[,1]),
           x1 = exp(aggdata[,1] + 2 * aggdata[,3]))
}

for (i in 1:274) {
  segments(y0 = exp(aggdata[,1]), y1 = exp(aggdata[,1]), x0 = exp(aggdata[,1]),
           x1 = exp(aggdata[,1] - 2 * aggdata[,3]))
}
```
